# A fatal case of disseminated pulmonary and renal mucormycosis caused by *Rhizopus microspores*

**DOI:** 10.1590/S1678-9946202365054

**Published:** 2023-10-20

**Authors:** Liuyang Hu, Xingchun Chen

**Affiliations:** 1 The People’s Hospital of Guangxi Zhuang Autonomous Region, Guangxi Academy of Medical Sciences, Department of Laboratory Medicine, Nanning, Guangxi, China

**Keywords:** Disseminated mucormycosis, Pulmonary mucormycosis, Renal mucormycosis, Diabetes mellitus

## Abstract

Rhinocerebral and pulmonary mucormycosis are the main manifestations of mucormycosis; however, disseminated pulmonary associated with renal mucormycosis is rarely reported. In this paper, we report a rare fatal case of disseminated pulmonary and renal mucormycosis caused by *Rhizopus microsporus* in a 50-year-old man with poorly controlled hypertension, type 2 diabetes, and prolonged use of corticosteroids for the treatment of his reiterative gouty arthritis. In this patient, the use of corticosteroids and poorly controlled diabetes were considered underlying risk factor for his disseminated mucormycosis, along with acute renal dysfunction, suggesting the need for clinical suspicion of disseminated pulmonary and renal mucormycosis in hospitalized patients with poorly controlled diabetes and immunocompromised host.

## INTRODUCTION

Mucormycosis is a rare life-threatening opportunistic infection characterized by rapid development^1^. The main risk factors of mucormycosis are diabetes mellitus, immunocompromised host, hematological malignancies, neutropenia, solid organ transplantation, autoimmune diseases, multiple injuries, and cortisol use^2^. The increasing prevalence of mucormycosis during the COVID-19 pandemic is becoming a growing concern^3^. Additionally, the role of diabetes and corticosteroid use an their relation with COVID-19-related Mucormycosis (CAM) was determined^3^. In recent years, there has been a growing number of reported cases of different types of mucormycosis, including rhinocerebral, cutaneous, pulmonary, cardiac, gastrointestinal, and disseminated; however, osteomyelitis, peritonitis, endocarditis, and renal infection are rare forms^4^. Species from *Rhizopus* genus are the most responsible for high-mortality mucormycosis^5^. *Rhizopus microspores* and *Rhizopus homothallicus* infections are rising in India^6^. The main methods used to diagnose Mucorales infections include direct KOH mount, special staining (such as GMS, PAS, and calcofluor white staining), as well as positive culture results from different clinical samples and histopathological demonstration. The Mucorales are usually fast-growing fungi characterized by primitive coenocytic, broad, and mostly aseptate hyphae. PCR testing and sequencing are reliable diagnostic techniques for Mucorales infections; however, these tests are limited, especially in low-income and middle-income countries.

In this study, we present a rare fatal case of disseminated pulmonary and renal mucormycosis caused by *Rhizopus microspores* in a 50-year-old man with poorly controlled hypertension, type 2 diabetes, and prolonged use of corticosteroids for the treatment of his reiterative gouty arthritis.

## CASE REPORT

**Figure 1:**
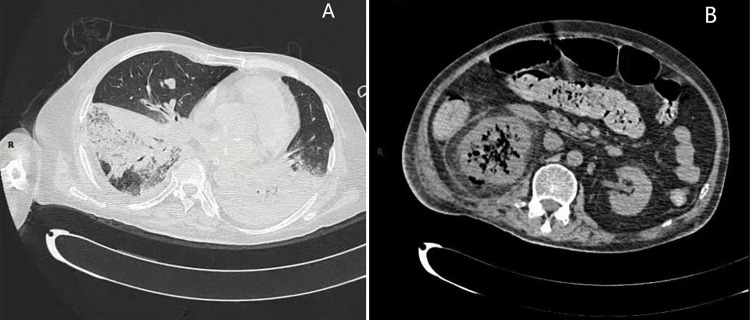
(A) Pulmonary CT showed bilateral patchy, cord-like high-density foci, and patchy ground glass shadows, with blurry edges and partial consolidation; (B) Abdominal CT revealed there was multiple gas in the right kidney surrounded by exudation shadows.

A 50-year-old rural man presented to the emergency department of the People’s Hospital of Guangxi Zhuang Autonomous Region, China, due to frequent cough, sneezing, recurrent fever, and expectoration for one month. Later, he was admitted to the emergency intensive care unit due to acute respiratory failure. He presented poorly controlled hypertension and type 2 diabetes, along with long-term prednisone use to treat his reiterative gouty arthritis for 10 years due to taking uric-acid-lowering drugs irregularly. The physical examination revealed fever, with 40 °C temperature peak, tachycardiac (116 beats/min), and blood pressure at 146/79mmHg. Laboratory results showed leukocytosis ( $20.21\times 10^{\wedge }9/L$ ), anemia (RBC: $2.43\times 10^{\wedge }12/L$ ; Hemoglobin: 73 g/L), thrombocytopenia ( $58\times 10^{\wedge }9/L$ ), C-reactive protein of >200.00 mg/L, Procalcitonin of >100.00 ng/mL, and poor glycemic control (Glu 23.2 mmol/L, HbA1c 9.7%). Other laboratory findings included abnormal urinalysis (leukocyte 3+, cell count 357 cells/μL; occult blood 2+, red blood cell count 124/µL ↑; glucose 3+; ketone body 1+; urinary protein+-). Pulmonary CT showed bilateral patchy, cord-like high-density foci, and patchy ground glass shadows, with blurry edges and partial consolidation (Figure 1A). Abdominal CT revealed the presence of multiple gas in the right kidney surrounded by exudation shadows (Figure 1B). He received oxygen inhalation and insulin pump hypoglycemic therapy after admission. The patient showed symptoms of shock and acute respiratory failure, and then underwent endotracheal intubation and mechanical-assisted ventilation, with continuous pumping of norepinephrine to maintain blood pressure and fluid infusion to improve circulation. A 0.5 mg of colchicine once a day, 5 mg of prednisone twice a day, and puncture injection of compound betamethasone into swollen and painful joints were administered to treat acute episodes of gouty arthritis. According to the distribution of pathogenic bacteria in the emergency care unit of the hospital, it was considered severe pneumonia caused by multidrug-resistant Gram-negative bacteria. *Enterococcus* specimen was detected in the blood culture of the patient’s first hospitalization in another hospital half a month before. Thus, broad-spectrum antibacterial therapy coverage with imipenem and daptomycin was used without determination of a clear infectious etiology after inter-consultation with the infectious disease team. However, there was no significant clinical improvement with antibacterial therapy.

**Figure 2:**
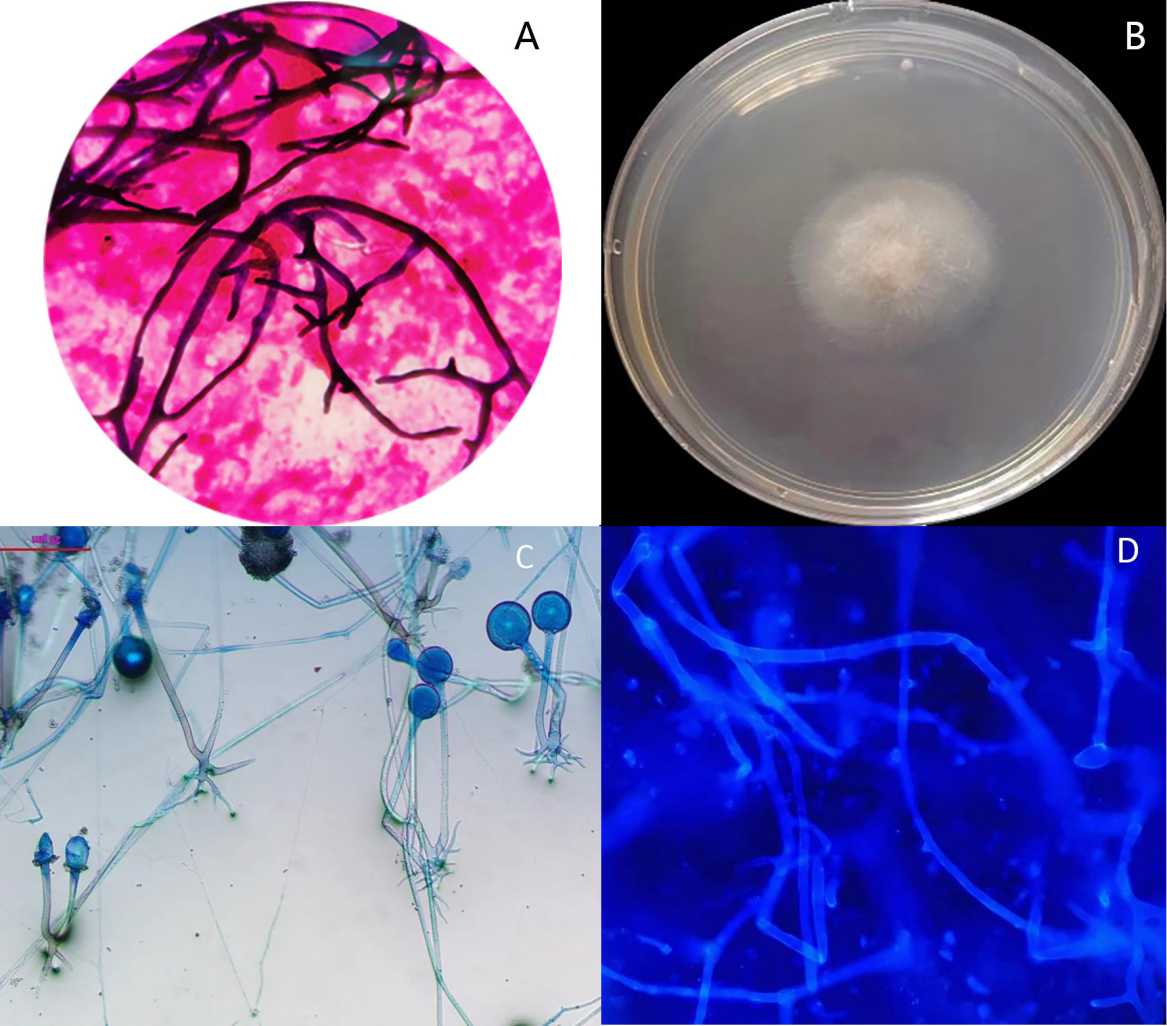
(A) Gram staining of BALF revealed wide, aseptate hyphae, ×1000; (B) Cotton-fluffy growth colony was isolated from BALF after 24-hour culture on Sabouraud dextrose agar; (C) Lactophenol-cotton blue stain showed stolons and pigmented rhizoids and opposite sporangiophores from nodes directly above the rhizoids, ×400; (D) Calcofluor white staining of urine revealed wide, aseptate hyphae, ×1000.

On the fourth day of admission, the multidrug-resistant *Enterobacteriaceae Buganda* (meropenem sensitive) was detected in culture from bronchial alveolar lavage fluid (BALF). Therefore, meropenem was used for *Enterobacteriaceae Buganda* pneumonia. On day 6 of admission, the Gram staining of BALF revealed wide, aseptate hyphae (Figure 2A). Cotton-fluffy growth colony was isolated from BALF after 24-h culture on Sabouraud dextrose agar (Figure 2B), which was suspected to be *Rhizopus* based on aseptate hyphae, characterized stolons, pigmented rhizoids, and opposite sporangiophores from nodes directly above the rhizoids seen on the lactophenol-cotton blue stain (Figure 2C). Internal transcribed spacer (ITS) sequencing was performed, and the obtained nucleotide sequence was compared with the nearest sequence at the NCBI GenBank database. The first homology sequence presenting the highest identity was the *Rhizopus microspores* rDNA region (97.99% nucleotide identity). On day 8 of admission, calcofluor white staining of urine revealed wide, aseptate hyphae (Figure 2D), and *Rhizopus microspores* was also isolated from his urine culture. Antifungal susceptibility testing was performed according to CLSI 38 methods, determining minimum inhibitory concentrations (MICs) after 24h of incubation for posaconazole (0.5 µg/mL), amphotericin B (2 µg/mL), both suggestive of susceptibility. Consequently, the antimicrobial treatments plan was revised to include meropenem for *Enterobacteriaceae Buganda* pneumonia, along with intravenous administration of 100 mg of amphotericin B cholesterol sulfate and nebulization of 10 mg twice a day for *Rhizopus microspores*. Unfortunately, the patient’s condition deteriorated the following day (day 9) and resulted in death.

## DISCUSSION

The incidence of mucormycosis have been increasing in patients with diabetes mellitus in recent decade, causing high mortality^7^. Rhinocerebral and pulmonary mucormycosis are the main manifestations of mucormycosis; however, disseminated pulmonary with renal mucormycosis is rarely reported^8^. Early clinical suspicion and accurate identification are crucial for managing mucormycosis due to its rapid invasion of blood vessels, resulting in high mortality rates. The ability of transferrin to transport iron is inhibited and the concentration of free iron is reduced in patients with diabetes, which is beneficial for the growth and reproduction of Mucor^9^. Pulmonary mucormycosis presents obvious dyspnea and hypoxemia^7^. Therefore, clinicians should have a high suspicion of pulmonary mucormycosis in diabetic patients who experience persistent dyspnea and hypoxemia that cannot be attributed to typical pneumonia. Renal mucormycosis is characterized by symptoms such as fever and flank pain resembling acute pyelonephritis, along with significant impairment of renal function, including elevated levels of blood creatinine and urea, proteinuria, hematuria, and pyuria^10^.

During the COVID-19 pandemic, the incidence rate of CAM increased significantly, especially in India^11^. The most significant risk factor of CAM was uncontrolled diabetes^3^. Patients with uncontrolled diabetes usually have an inflammatory state, which can be enhanced by activating the antiviral immunity against SARS-CoV-2, which may be conducive to secondary infection^3^. The increase in cases of CAM has been associated with continuous use of corticosteroids^3^. A recent report indicated that the incidence rate of mucormycosis would increase even if patients with diabetes were treated with corticosteroids for a short time^12^. One report has also mentioned that the patients who received a cumulative dose exceeding 600 mg of prednisone or 2-7 g of methylprednisolone are more prone to mucormycosis^12^. Corticosteroids are the most commonly used drugs to treat COVID-19, leading to a hyperglycemic state in diabetes patients, which provide a fertile soil for Mucorales fungi^12^. The coexistence of COVID-19 infection, diabetes, and corticosteroid use contributed to the disruption of patients’ immune systems, resulting in the development of invasive mucormycosis^12^. If a patient with COVID-19 is suspected or confirmed to have mucormycosis, all immunosuppressive treatments should be reduced or discontinued based on the clinical condition^12^.

Combining medical drug treatment with active surgical treatment and optimizing the patient’s conditions, such as correcting acidosis, correcting hyperglycemia, restoring the white blood cell count, and adjusting immunosuppressants use, can significantly improve the survival rate of patients^13^. Liposome amphotericin B should be used as a first-line treatment drug and administered early. Delayed initiation of liposome amphotericin B therapy (>6 days after diagnosis) has been associated with doubling mortality rate at three months^14^. Isavuconazole has recently become a first-line alternative for patients who are either intolerant to amphotericin B or do not respond favorably to it^15,16^. Moreover, it has been documented that the all-cause mortality of isavuconazole is similar to that of liposome amphotericin B in a limited number of patients as first-line treatment (weighted all-cause mortality: 33% vs 41%; p=0·595)^17^. Some cases of successful treatment of mucormycosis with posaconazole have been documented^18,19^, although it is currently believed that posaconazole can only be considered as a second-line treatment or salvage treatment^20^. The empirical use of active antifungal drugs for mucormycosis is reasonable in patients with clear risk factors, but routine use of posaconazole for mucormycosis prevention remains controversial, which will increase the risk of fungal resistance to posaconazole. Furthermore, adjunctive therapy, such as iron chelators, statins, GM-CSF, colistin and cytokines, shows potential to improve outcomes^13^.

## CONCLUSION

Briefly, in this case, the primary risk factors for the patient’s disseminated mucormycosis were the use of corticosteroids and poorly-controlled diabetes. It is uncommon to see disseminated pulmonary with renal mucormycosis. Mortality might be reduced by improved clinical suspicion, early initiation of antifungals, and timely diagnosis, along with aggressive surgical debridement of necrotic lesions and reversal of underlying immunosuppression.
